# Assessing the treatment of cannabidiolic acid methyl ester: a stable synthetic analogue of cannabidiolic acid on *c*-Fos and NeuN expression in the hypothalamus of rats

**DOI:** 10.1186/s42238-021-00081-1

**Published:** 2021-07-12

**Authors:** Eric Murillo-Rodríguez, Diana Millán-Aldaco, Gloria Arankowsky-Sandoval, Tetsuya Yamamoto, Roger G. Pertwee, Linda Parker, Raphael Mechoulam

**Affiliations:** 1 Laboratorio de Neurociencias Moleculares e Integrativas Escuela de Medicina, División Ciencias de la Salud, Universidad Anáhuac Mayab Mérida, Km. 15.5 Carretera Mérida-Progreso, Int. Km. 2 Carretera a Chablekal, Yucatán C.P. 97,308 Mérida, México; 2 Intercontinental Neuroscience Research Group, Mérida, Yucatán México; 3 Depto. de Neurociencia Cognitiva. División de Neurociencias, Instituto de Fisiología Celular, Universidad Nacional Autónoma de México, Ciudad de México, México; 4 Centro de Investigaciones Regionales “Dr. Hideyo Noguchi”, Universidad Autónoma de Yucatán, Mérida, Yucatán México; 5 Graduate School of Technology, Industrial and Social Sciences, Tokushima University, Tokushima, Japan; 6 School of Medicine, Medical Sciences and Nutrition, Institute of Medical Sciences, University of Aberdeen, Aberdeen, UK; 7 Department of Psychology and Neuroscience Graduate Program, University of Guelph, Guelph, Ontario Canada; 8 Institute for Drug Research, Medical Faculty, Hebrew University, Jerusalem, Israel

**Keywords:** Cannabis, Hypothalamus, Rat, Sleep, Wakefulness

## Abstract

**Background:**

Cannabidiol (CBD), the non-psychotropic compound from *Cannabis sativa,* shows positive results on controlling several health disturbances; however, comparable data regarding additional chemical from *C. sativa,* such as cannabidiolic acid (CBDA), is scarce due to its instability. To address this limitation, a stable CBDA analogue, CBDA methyl ester (HU-580), was synthetized and showed CBDA-like effects. Recently, we described that HU-580 increased wakefulness and wake-related neurochemicals.

**Objective:**

To extend the comprehension of HU-580´s properties on waking, the *c*-Fos and NeuN expression in a wake-linked brain area, the hypothalamus was evaluated.

**Methods:**

*c*-Fos and NeuN expression in hypothalamic sections were analyzed after the injections of HU-580 (0.1 or 100 μg/kg, i.p.).

**Results:**

Systemic administrations of HU-580 increased *c*-Fos and neuronal nuclei (NeuN) expression in hypothalamic nuclei, including the dorsomedial hypothalamic nucleus dorsal part, dorsomedial hypothalamic nucleus compact part, and dorsomedial hypothalamic nucleus ventral part.

**Conclusion:**

HU-580 increased *c*-Fos and NeuN immunoreactivity in hypothalamus nuclei suggesting that this drug might modulate the sleep–wake cycle by engaging the hypothalamus.

## Background

Several pieces of evidence have suggested that the non-psychotropic molecule derived from *Cannabis sativa,* cannabidiol (CBD), exerts positive therapeutic pharmacological properties for the management of several health disturbances, including epilepsy, pain, anxiety, among many others (Fraguas-Sánchez and Torres-Suárez, 2018; Friedman and Wongvravit, 2018; Millar, et al. 2019; Premoli, et al. 2019; Pretzsch, et al. 2019). However, only limited experimental data is available concerning the effects of another molecule from *C. sativa*, cannabidiolic acid (CBDA). The lack of evidence of this cannabinoid lies in its chemical instability (Citti, et al. 2018; Mechoulam and Hanus, 2002). Hence, to tackle this problem, our group has synthetized a stable CBDA analogue named CBDA methyl ester of HU-580, which produces certain CBDA-like effects more potently than CBDA. These pharmacological properties of HU-580 include the management of anxiety and depression in experimental models (Hen-Shoval, et al. 2018; Pertwee, et al. 2018). In addition, HU-580 modulates the sleep–wake cycle by increasing wakefulness as well as wake-related neurochemicals such as dopamine, serotonin, adenosine, and acetylcholine (Murillo-Rodríguez et al. 2020). Despite these fascinating results, the mechanism of action activated by HU-580 for modulation of the sleep–wake cycle is unknown. Therefore, to provide further evidence of the neurobiological effects of HU-580 on sleep control, we evaluated whether administrations of this chemical might induce changes on the expression of neural markers, such as *c*-Fos and neuronal nuclei (NeuN), in the hypothalamus, a brain region that has been linked to the regulation of wakefulness (Aston-Jones et al. 2001; Chen, et al. 2018; Saper et al. 2005; Sapin et al. 2010).

## Methods

### Ethics

All experimental procedures were performed in accordance with the Research and Ethics Committees of our Institution and met the guidelines of Mexican Standards Related to Use and Management of Laboratory Animals (DOF. NOM-062-Z00-1999), fulfilling the ARRIVE guidelines in accordance with the U.K. Animals (Scientific Procedures; Act, 1986 and associated guidelines, EU Directive 2010/63/EU for animal experiments) as well as the National Institute of Health (NIH publication No. 80–23, revised 1996 and Guide for the Care and Use of Laboratory Animals, 8th edition, 2011).

### Animals

Male Wistar rats (*N* = 15; 250–300 g) were singly housed in transparent acrylic cages (48 × 20 × 27 cm) with standard bedding material, chow pellets (Purina Rat Chow, México), and tap water ad libitum. Experimental conditions included housing all rats at 12-h light/dark cycle (lights on at 07:00 h; 200 lx), controlled temperature (22 ± 1 °C), and relative humidity (60 ± 10%). All efforts were made to minimize animal suffering and using the minimal number of animals required to produce reliable results.

### Chemicals

HU-580 was synthetized by our group as previously described and prepared in a vehicle (VEH) solution (Hen-Shoval, et al. 2018; Pertwee, et al. 2018). Paraformaldehyde, phosphate-buffered saline (PBS), sucrose, glycerol, dimethyl sulfoxide (DMSO), solvents, and chemicals were purchased from Sigma-Aldrich (St. Louis, MO, USA) or elsewhere. Reagents for immunohistochemical studies were obtained from Santa Cruz Biotechnology, Inc. (Dallas, TX, USA), Millipore (Billerica, MA, USA), and Vector Laboratories (Burlingame, CA, USA).

### Experimental design

The rats were assigned randomly to one of two treatment conditions: vehicle (1 mL/i.p.; *n* = 5) or HU-580 (0.1 or 100 μg/kg/1 mL; i.p.; *n* = 5; each dose). To avoid circadian influences on the expression of *c*-Fos or NeuN, all systemic administrations were given 1 h after the beginning of the lights-on period. In addition, we used a single-blind study in which members of the laboratory that applied the administrations were not aware about the code of the treatments.

### Brain tissue collection

One hour after the treatments were applied, rats were sacrificed by a lethal dose of pentobarbital (150 mg/kg; i.p.) and perfused intracardially with sodium chloride (0.9%) followed by paraformaldehyde (4.0%; Sigma-Aldrich, St. Louis, MO, USA) in PBS (0.1 M, pH 7.1) as previously described (Macías-Triana et al. 2020). Staff members of the laboratory blinded to the code of rats developed the perfusion in all rats. Later, the brains were removed, post-fixed in the same fixative solution overnight at 4 °C and then equilibrated following previous procedures by sucrose immersion (10, 20, or 30% sucrose/0.1 M PBS during 24 h each concentration or until tissue sinks). After complete equilibration by infiltration of the sucrose, the brains were cut in coronal Sects. (20 μm thickness) and collected in 1:5 serial order using a Portable Bench-top Cryostat (Leica CM1100. Leica Microsystems GmbH. Wetzlar, Germany; Macías-Triana et al. 2020). To avoid experimental bias, member of the laboratory blinded to the code of rats cut the brains. Due to the hypothalamus has been linked with wakefulness control (Heiss et al. 2018; Latifi et al. 2018; Naganuma et al. 2019), this brain area was chosen for the immunohistochemical study. The identification of the hypothalamic nuclei, including the dorsomedial hypothalamic nucleus dorsal part (DMD), dorsomedial hypothalamic nucleus compact part (DMC), and dorsomedial hypothalamic nucleus ventral part (DMV) was done by the aid of the Rat Brain Atlas which included coordinates from − 2.28 to − 3.48 mm (from Bregma according the Rat Brain Atlas (Paxinos and Watson, 2005). Once collected, the sections were stored in cryoprotective solution (glycerol [20%] and DMSO [2%] in sodium phosphate [0.1 M]) at – 20 °C (Thermo Fisher Scientific Revco, Waltham, MA, USA). The whole brain collection procedures were developed as previously reported (de-la-Cruz et al. 2018).

### c-Fos and NeuN immunohistochemical analysis

Since the immediate early gene c-fos (Chung, 2015; Kovács, 2008) and NeuN (Duan et al. 2016; Gusel'nikova and Korzhevskiy, 2015) have long been known as molecular markers of neuronal activity, then the expression of these proteins was addressed in DMD, DMC, and DMV in control and HU-treated animals. In detail, slides from control and HU-580 groups (0.1 or 100 μg/kg; i.p.) were prepared for *c*-Fos and NeuN immunohistochemical analysis using standardized procedures as previously described (Ni et al. 2020; Plaisier et al. 2020). Serial coronal cryostat sections of the DMD, DMC, and DMV were processed for *c*-Fos and NeuN immunoreactivity, imaged, and quantified as described previously (de-la-Cruz et al. 2018). The slides were washed 3 times in phosphate-buffered saline (PBS; 0.1 M, pH 7.3) and later to inactivate the endogenous peroxidase, the sections were incubated in periodic acid (0.28%) during 1 min at room temperature, with hydrogen peroxide (H_2_O_2_; 3%) and methanol (10%) in PBS (0.1 M) for 20 min at room temperature. Next, the slides were washed 3 times in PBS (0.1 M, pH 7.3) and blocked with donkey or goat serum (10%) diluted in PBS (containing 0.2% Triton X-100. Sigma-Aldrich, St. Louis, MO, USA). Subsequently, the slides were incubated with the corresponding primary antibody at 4 °C (Goat anti-c-fos 1:100; Santa Cruz Biotechnology, Inc. Dallas, TX, USA) or mouse anti-NeuN (1:500; Millipore. Billerica, MA, USA) overnight. On the next day, the slides were again washed 3 times in PBS (0.1 M, pH 7.3) and incubated for 2 h at room temperature with the respective biotinylated secondary antibody (1:250 dilution, goat anti-mouse IgG; Sigma-Aldrich, St. Louis, MO, USA. Donkey anti-rabbit IgG; Vector Laboratories. Burlingame, CA, USA). Upon the application of the secondary antibody, the slides were washed another 3 times in PBS (0.1 M, pH 7.3) and incubated with the peroxidase complex (1:2000. Sigma-Aldrich, St Louis, MO, USA) for 1 h in a dark room. Lastly, following 3 washes in PBS, the immunoreactivity was revealed by exposing the sections to diaminobenzidine (0.05%; Sigma-Aldrich. St Louis, MO, USA) and H_2_O_2_ (0.03%) in PBS. The reaction was stopped using PBS and slides were then washed several times in PBS again. Once immunoreactivity was achieved, all slides were mounted onto chrome alum gelatin-coated slides, dehydrated through graded alcohols, cleared in xylene and cover slipped with histology slide mounting medium (DPX Mountant, Sigma-Aldrich, St Louis, MO, USA). To confirm the reproducibility of the immunohistochemical experiments, batches containing approximately the same number of slides from the experimental groups were stained using the same primary antibody simultaneously whereas the negative controls included slides analyzed under an identical immunohistochemical procedure with the exception that 1% bovine serum albumin in PBS was substituted for the primary antibody. One observer blind to the experimental codes of slides developed the *c*-Fos and NeuN immunohistochemistry.

### Imaging and image analysis of c-Fos- and NeuN-positive neurons

A Rat Brain Atlas (Paxinos and Watson, 2005) was used as a reference to identify the *c*-Fos and NeuN labeled neurons in DMD, DMC, and DMV. Immunoreactivity was visualized with an Axio Imager Microscope (A2m, Carl Zeiss AG, Oberkochen, Germany) with an attached microscope camera (AxioCam, Carl Zeiss AG, Oberkochen, Germany). The images were acquired using a computerized image analysis system ZEN (Blue Edition, Carl Zeiss AG, Oberkochen, Germany). A laboratory staff member blinded to the code of all slides, counted the *c*-Fos- and NeuN-positive immunostaining as previously reported (de-la-Cruz et al. 2018).

### Statistical analysis

Using StatView software (version 5.0.0, SAS Institute, USA), data were analyzed by one-way analysis of variance (ANOVA) applied with multiple comparisons using the Scheffé’s post hoc analysis. Differences between groups were considered statistically significant at values of *P* < 0.05. Results are expressed as mean ± S.E.M. For investigating the relationship among the HU-580 doses and *c*-Fos and NeuN expression, Pearson’s correlation coefficient (*r*) was used (StatView; version 5.0.0, SAS Institute, USA). The strength of association between these variables was established if *r* ≥ 0.6 and *P* < 0.05. In addition, linear regression analysis (*R*^*2*^) was used to test if the dosage of HU-580 (0.1 or 100 μg/kg; i.p.) significantly would predict the increase the number of positive *c*-Fos and NeuN neurons. Significant statistical values for *R*^*2*^ were determined within the range of 0–1 and *P* < 0.05.

## Results

### Expression of c-Fos and NeuN immunoreactivity in the hypothalamic nuclei in response to HU-580

**Fig. 1:**
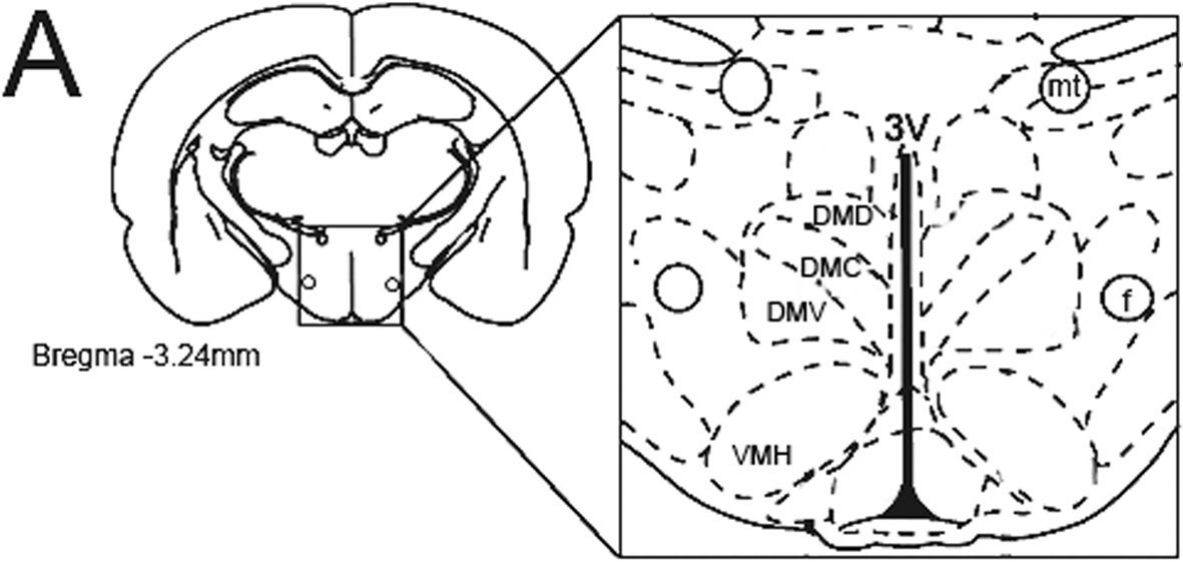
The schematic illustration from the rat brain atlas (Paxinos and Watson, 2005) showing the hypothalamus section taken for the immunohistochemical studies (Panel **A**). A representative illustration depicting the location of the relative density of *c*-Fos and NeuN expression in the hypothalamus. Drawing obtained from Paxinos and Watson's Atlas (2005)

To test whether HU-580 promoted changes in *c*-Fos and NeuN expression, we analyzed the immunohistochemical staining in the hypothalamic nuclei including the DMD, DMC, and DMV. Figure 1A displays a representative illustration depicting the location of the relative density of *c*-Fos and NeuN immunoreactivity in the targeted areas. As shown for *c*-Fos analysis, and compared to control (Fig. 1B), systemic injections of HU-580 (0.1 or 100 μg/kg; i.p.; Fig. 1C, D, respectively) increased *c*-Fos expression in DMD, DMC, and DMV. Moreover, compared to the control group (Fig. 1E), similar findings were observed in NeuN expression in rats treated with HU-580 (0.1 or 100 μg/kg; i.p.; Fig. 1F, G, respectively).

### Number of c-Fos-positive neurons in the hypothalamic nuclei in response to HU-580

**Fig. 2:**
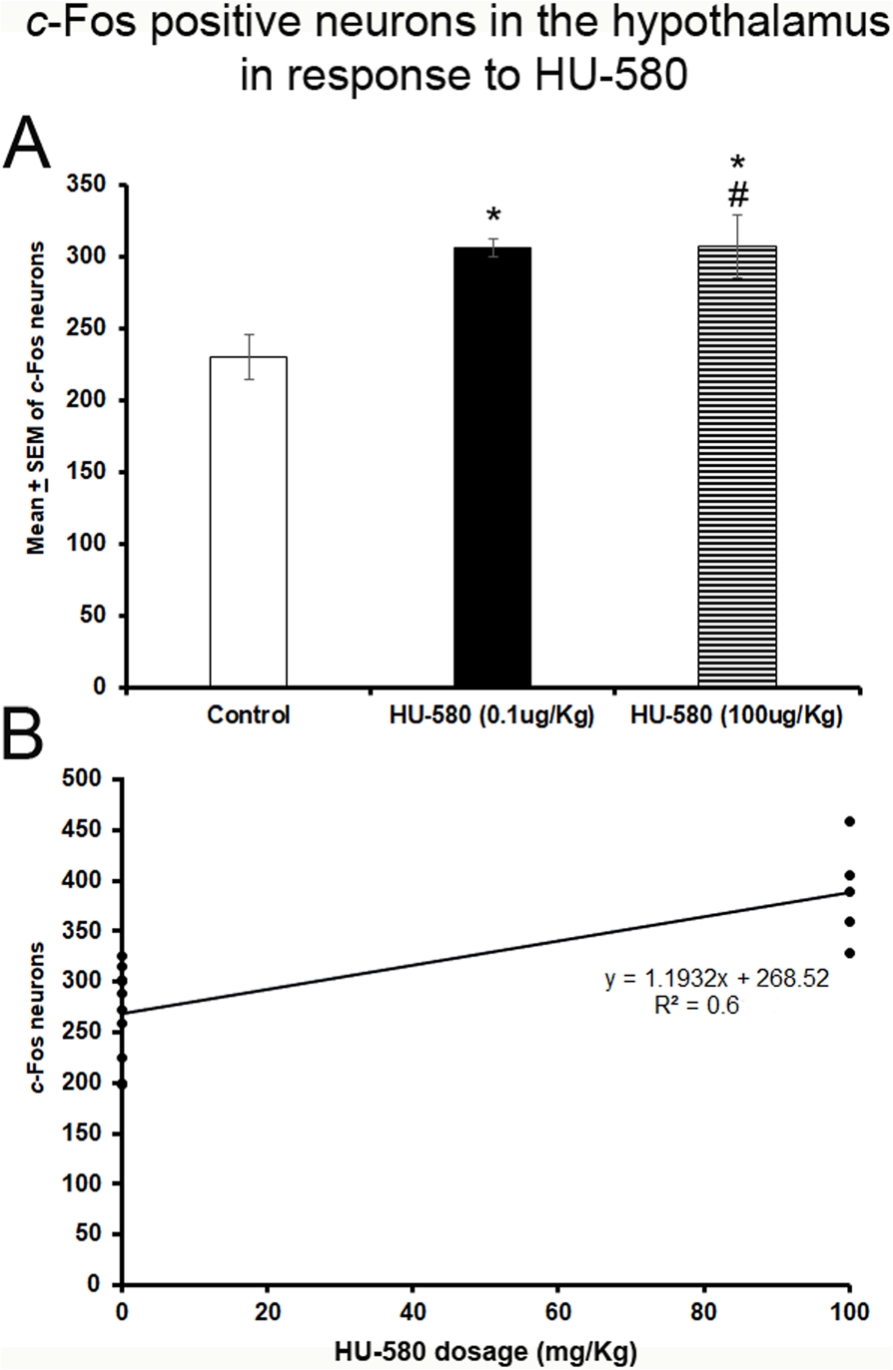
The number of *c*-Fos-positive neurons in the hypothalamic nuclei in response to HU-580. **A** The significant increase in *c*-Fos expression in hypothalamus nuclei from HU-treated rats ((0.1 or 100 μg/kg; i.p.) as compared to controls (*F*_*(2, 12)*_ = 24–738; *P* < 0.0001). The post hoc analysis showed significant differences among the experimental data (Scheffé’s post hoc test: control vs. HU-580 (0.1 μg/kg, *P* < 0.01; control vs. HU-580 (100 μg/kg), *P* < 0.0001; HU-580 (0.1 μg/kg) vs. HU-580 (100 μg/kg), *P* < 0.01). **B** The Pearson’s correlation coefficient analysis with a significant and positive relationship between the used doses of HU-580 (0.1 or 100 μg/kg; i.p.) and the Fos immunoreactivity (*r* = 0.6, *P* < 0.0002). Finally, the linear regression analysis showed that administrations of different doses of HU-580 predicted the enhancement in the number of Fos expression in the hypothalamic nuclei (*R*^2^ = 0.6, *P* < 0.0005; **B**)

HU-treated (0.1 or 100 μg/kg; i.p.) rats showed a significant increase in the number of Fos-positive neurons in the hypothalamic nuclei as compared to control group (*F*_(2, 12)_ = 24.738; *P* < 0.0001; Fig. 2A). Further post hoc analysis showed significant differences between experimental treatments (Scheffé’s post hoc test: control vs. HU-580 (0.1 μg/kg), *P* < 0.01; control vs. HU-580 (100 μg/kg), *P* < 0.0001; HU-580 (0.1ug/Kg) vs. HU-580 (100 μg/kg), *P* < 0.01).

Our next result, from the Pearson’s correlation coefficient analysis, showed a significant and positive relationship between the tested doses of HU-580 (0.1 or 100 μg/kg; i.p.) and the Fos immunoreactivity (*r* = 0.6, *P* < 0.0002; Fig. 2B). Current findings suggest a significant dose-dependent interaction between HU-580 and *c*-Fos expression in hypothalamic nuclei. In regard to the linear regression analysis, we fund that HU-580 (0.1 or 100 μg/kg; i.p.) significantly would predict the enhancements on quantitative Fos expression. Thus, administrations of different doses of HU-580 predicted the increase in the number of Fos immunoreactivity in hypothalamic nuclei (*R*^2^ = 0.6, *P* < 0.0005; Fig. 2B). We conclude that as higher doses of HU-580 were administered, higher Fos expression was found in hypothalamic nuclei.

### Number of NeuN-positive neurons in the hypothalamic nuclei in response to HU-580

**Fig. 3:**
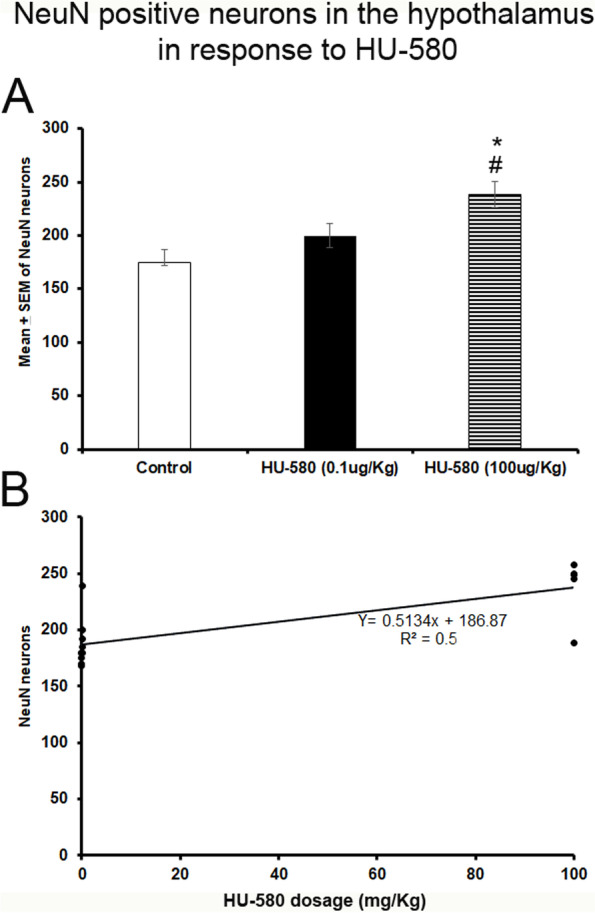
The number of NeuN positive neurons in the hypothalamus in response to HU-580. Systemic injections of the highest dose of HU-580 (100μg/Kg) increased the number of NeuN positive immunoreactive neurons in the hypothalamus (Panel **A**; *F*_(2, 12)_= 11.334; *P*< 0.001). The Scheffé´s post hoc test displayed significant differences between the experimental groups for NeuN expression in the hypothalamus (Control vs. HU-580 (0.1μg/Kg), *P*= 0.2; Control vs. HU-580 (100μg/Kg), *P*< 0.001; HU-580 (0.1μg/Kg) vs. HU-580 (100μg/Kg), *P*< 0.04). The Pearson’s correlation coefficient analysis among the doses of HU-580 (0.1 or 100μg/Kg; i.p.) and the NeuN expression showed a significant and positive relationship between these experimental variables (*r*= 0.5, *P*< 0.0008; Panel **B**) whereas the linear regression analysis indicated that HU-580 (0.1 or 100μg/Kg; i.p.) produced a significantly increase in quantitative NeuN neuronal expression in the hypothalamus (*R*^2^= 0.5, *P*< 0.001)

In regard to the effects of HU-580 on NeuN expression, we found a significant increase in this molecular marker in rats that received a systemic injections of HU-580 (0.1 or 100 μg/kg) compared to control group (*F*_(2, 12)_ = 11.334*; P* < 0.001; Fig. 3A). The Scheffé’s post hoc test displayed significant differences among the experimental trials for NeuN immunoexpression in the hypothalamic nuclei (control vs. HU-580 (0.1 ug/kg), *P* = 0.2; control vs. HU-580 (100 μg/kg), *P* < 0.001; HU-580 (0.1 μg/kg) vs. HU-580 (100 μg/kg), *P* < 0.04). Regarding the Pearson’s correlation coefficient analysis among the doses of HU-580 (0.1 or 100 μg/kg; i.p.) and the NeuN expression, a significant and positive relationship between these experimental variables was found (*r* = 0.5, *P* < 0.0008; Fig. 3B). Therefore, data suggest that significant interactions among the different doses of HU-580 and NeuN activity in hypothalamic nuclei were present. In addition, the linear regression analysis indicated that HU-580 (0.1 or 100 μg/kg; i.p.) produced a significantly dose-related increase in quantitative NeuN neuronal expression in hypothalamic nuclei (*R*^2^ = 0.5, *P* < 0.001; Fig. 3C). We conclude that higher doses of HU-580 promote higher NeuN expression in hypothalamic nuclei.

## Discussion

Limited research has revealed the pharmacological properties of cannabidiolic acid (CBDA), a constituent of *Cannabis sativa*. However, CBDA is rather unstable (Chou et al. 2003; Citti et al. 2018; Crombie and Crombie, 1977), suggesting that its chemical instability proved difficult and need further studies. To address this issue, a stable analogue of CBDA named CBDA methyl ester (HU-580) was recently synthesized showing greater potency than CBDA at, for example, producing apparent anxiolytic and antidepressant effects in vivo (Hen-Shoval, et al. 2018; Pertwee, et al. 2018). To gain knowledge regarding the pharmacological profile of HU-580 on neurobiological functions, we have published that systemic injections of this compound induced wake-promoting effects accompanied by enhancements in wake-related neurochemicals such as dopamine, adenosine, and acetylcholine (Murillo-Rodríguez et al. 2020). These fascinating findings prompted a need to identify the putative neuroanatomical substrate involved in HU-580-induced sleep modulation. Thus, here we have demonstrated that systemic injections of HU-580 (0.1 or 100 μg/kg; i.p.) promoted neuronal activation as determined by *c*-Fos and NeuN immunohistochemical assays. Under our conditions, HU-580 enhanced *c*-Fos and NeuN expression in hypothalamic nuclei comprising dorsomedial hypothalamic nucleus dorsal part (DMD), dorsomedial hypothalamic nucleus compact part (DMC), and dorsomedial hypothalamic nucleus ventral part (DMV). The data we have obtained suggest that HU-580 might exert wake-promoting effects via the engagement of neuronal activity located in DMD, DMC, and DMV. Even though the mechanism of action of HU-580 underlying its regulation of wakefulness has not been discovered yet, we would like to draw the following hypothetical frame: HU-580 seems to induce neuronal activity evaluated by *c*-Fos and NeuN immunoreactivity in hypothalamic nuclei which has been suggested as modulator of wakefulness (Aston-Jones et al. 2001; Chen, et al. 2018; Saper et al. 2005; Sapin et al. 2010). Further studies support our hypothetical frame in regard the likely engagement of hypothalamic nuclei in the wake-promoting effects of HU-580 since current evidence shows that prolonged wakefulness induces an increase in *c*-Fos expression (Azeez et al. 2018).

### Limitations of the study

Indeed, we recognize several limitations of our findings as follows: (i) the *c*-*Fos* study lacks the characterization of certain neuronal types. Moreover, despite that Fos shows a fast and transient induction curve in activated neurons (Kim, et al. 2019) and the half-life of this protein is ~ 40–60 min (Kovács, 2008; Stancovski et al. 1995), the activity of Fos is not strictly correlated with neuronal activity (Cirelli and Tononi, 2000; Ito et al. 2005); (ii) some additional neuronal populations might be involved in HU-580’s effects. For instance, DMD sends rostral afferents to the ventrolateral preoptic nucleus (Deurveilher et al. 2002; Lu et al. 2001), a region in which lesions cause insomnia (Gvilia, 2010; Lüthi, 2019; Peyron, et al. 1998). Therefore, it is likely the engagement of the ventrolateral preoptic nucleus in HU-580’s effects; (iii) to advance the current comprehension of the mechanism underlying the effects of HU-580 on c-Fos and NeuN expression, it will be necessary to determine the identity of the responding neurons to HU-580 and to understand how the drug activates these neurons. Since the hypothalamic nuclei also projects to the lateral hypothalamic area which many neurons contain the wake-promoting neuropeptide hypocretin also known as orexin (Arrigoni et al. 2019; Backholer et al. 2009; Chen et al. 2018; Eyigor, et al. 2012; Nollet et al. 2011; Ono and Yamanaka, 2017; Peyron and Kilduff, 2017; Sapin et al. 2010; Sakurai, et al. 1998; Tyree et al. 2018; Wang, et al. 2018), it is highly possible that neurons reacting to HU-580 might be hypocretinergic; (iv) whether HU-580 modulates neurons located in hypothalamic nuclei will require further study by using alternative experimental approaches such as electrophysiological recordings, double-staining, or optogenetic procedures. The study, in its present form, is very limited in scope; however, it provides, for the very first time, that HU-580 exerts effects on *c*-Fos and NeuN expression in hypothalamus.

## Conclusions

The new pharmacological data we have now obtained suggest that HU-580 can enhance the expression of *c*-Fos and NeuN activity in hypothalamus, a brain area related to the regulation of wakefulness. The results obtained in this investigation allow to conclude that HU-580 might engage hypothalamic nuclei activity in rats for regulation of wakefulness. Indeed, further studies are still required to determine the mechanism of action that underlies the sleep–wake cycle effects of HU-580.

## Data Availability

The datasets generated during and/or analyzed during the current study are available from the corresponding author on reasonable request.

## Abbreviations

ANOVA: Analysis of variance
CBD: Cannabidiol
CBDA: Cannabidiolic acid
DMC: Dorsomedial hypothalamic nucleus compact part
DMD: Dorsomedial hypothalamic nucleus dorsal part
DMOS: Dimethyl sulfoxide
DMV: Dorsomedial hypothalamic nucleus ventral part
HU-580: CBDA methyl ester
PBS: Phosphate-buffered saline
*r*: Pearson’s correlation coefficient
*R*^*2*^: Linear regression analysis
VEH: Vehicle
